# Team diversity, conflict, and trust: Evidence from the health sector

**DOI:** 10.3389/fpsyg.2022.935773

**Published:** 2022-10-10

**Authors:** Muhammad Rafay Nawaz, Muhammad Ishtiaq Ishaq, Rehan Ahmad, Muhammad Faisal, Ali Raza

**Affiliations:** ^1^Institute of Quality and Technology Management, University of the Punjab, Lahore, Pakistan; ^2^School of Management Sciences, Quaid-i-Azam University, Islamabad, Pakistan; ^3^Imperial College of Business Studies, Lahore, Pakistan; ^4^COMSATS Institute of Information Technology, Islamabad, Pakistan

**Keywords:** diversity, conflict, trust, teams, healthcare industry, Pakistan

## Abstract

The current study aims to determine the impact of diversity and intra-team trust on conflict within the health sector of Pakistan. This study also measures the moderating role of trust in the relationship between diversity and conflict among team members. Data was collected using personally administered questionnaires from 61 teams, including 377 respondents working in 4 public sector hospitals in Pakistan, which were selected using a simple random sampling technique. The results revealed that diversity (as a composite) positively influences task conflict, while its two components—surface-level diversity and deep-level diversity—are associated positively with task conflict. Moreover, the results also lead to an exciting finding that trust among team members could reduce the positive influence of diversity on team members’ conflict. The implications for theory and practitioners are presented along with the avenues for future research directions.

## Introduction

Today’s turbulent and ever-changing economies present enormous challenges for all kinds of businesses to succeed, With success of course depending on how well a respective business performs. An increasing trend among organizations to use team-based structures. Task arrangement for teams can produce a responsive and flexible style to handle the current dynamics of today’s challenging environment (Hitt et al., 2001; Hollenbeck et al., 2004; Bell, 2007; Dolmans et al., 2015). The trend of using team-based structures has increased over time. Various models have been established to understand team effectiveness, and team composition is a critical constituent in most models (Tasheva and Hillman, 2018). In the composition of those teams, the articulation of diversity as a feature has attained considerable attention from researchers who consistently report its sterling impact on the effectiveness of team outcomes (Hjerto and Kuvaas, 2017; Ng et al., 2017).

Delegating responsibility and assembling people as a team for a job usually leads to challenging confrontations. A number of researchers (Hollenbeck et al., 2012; Costa et al., 2015; Li et al., 2016; Qu, 2017) claimed that individuals often have diverse viewpoints on how to handle a particular situation, but interdependent action certainly leads toward intra-team conflict. Moreover, the concept of diversity, which is defined here as combining the knowledge, skills, and abilities of multiple team members from different backgrounds and genders to handle work assignments will benefit an organization (Marks et al., 2001; Hawkins, 2016; Mor Barak, 2016). Bamel et al. (2018) proposed that intra-team conflicts may be avoided if an organization used knowledge, skills, and abilities more systematically.

Existing literature contains evidence of investigating those aspects influencing progress to improve organizational performance in all fields. Conflict as a variable has been discussed in the literature; although one facet of conflict is sometimes declared as beneficial (de Wit et al., 2012; Behfar et al., 2016; Held, 2017) it is mainly reported to seriously harm the performance of an organization by obstructing decision making and/or by impeding consensus and acceptance of decisions made (Abott, 2010; Hu et al., 2019). Such conflict and friction among team members regarding work leads them to blame each other instead of focusing on work. Accusations and blame between coworkers hinder goal-achievement and damage intra-team working relationships. Hence, they must be resolved (Jiang et al., 2016).

This study conducted a preliminary investigation and learned that conflict is rarely examined in the health sector of Pakistan. Moreover, the study targets the health sector because of the contextual likeliness of the work environment of the health sector of Pakistan being conducive to instigating conflict. Because of the high stakes of medical decisions, the role of trust among team members becomes more crucial (Olson et al., 2007). In these work environments, people have a specialized but different set of knowledge and skills in the same team, making it diverse. Employees’ work and tasks are highly interdependent; they have to face crucial challenges in emergencies, which may cause a stressful working climate, and they have to deal with other human beings in their work; hence conflicts are common in patient care because of their sensitivity, and their resolution is critical (Olson et al., 2007; Baker et al., 2017; Löhr et al., 2017). Managing conflict in an interdependent culture like that of Pakistan becomes a complicated endeavor. Therefore, this study intends to identify and clarify complications regarding conflict management in Pakistan.

It is essential to examine what may cause such conflicts in order to resolve them. Since differences among individuals are the roots of conflict, conflict is more likely to develop in diversified work teams. Organizations intentionally establish diversity in teams because its reported effectiveness (Guzzo and Dickson, 1996; Li et al., 2018) makes it imperative to include diversity in work teams. The insertion of diversity at a wider scale urges the researchers to study it from different perspectives, especially when scrutinizing the significant causes of conflict and its management in work teams. The existing literature provides a model that depicts the relationship between team diversity and conflict with trust as a moderating factor. Trust, when studied individually, has been classified into two categories that are not necessarily mutually exclusive. Similarly, the literature is rich with studies on two types of conflicts that can exist simultaneously and such that one can lead to another. However, any congruence among the individual categories of each construct can be seen as a deficit in the existing literature.

## Literature review

Past literature preserves the reported evidence on an increasing trend among organizations to use team-based structures. The Hawthorne studies in the 1920s were among the initial studies to feature the importance of teams (Campion et al., 1996; Muldoon, 2017). Since then, practitioners and researchers have strived to understand and clarify the factors influencing team processes and outcomes. Lee and Yu (2004) also noted more than three decades ago that “small teams are, quite simply, the basic organizational building blocks of excellent companies.” The trend of using team-based structures has increased ever since. Currently, most organizations employ team-based structures, which proved to be a keystone for effectiveness in organizations. More recently, researchers are venturing more time and effort into developing a more comprehensive model for the effectiveness of teams (Jules, 2007; Pretty et al., 2009; Scott et al., 2017; Rego et al., 2019; Selzer et al., 2021).

Some models are already being offered to clarify team effectiveness (e.g., McGrath, 1964; Zhang et al., 2015; Breuer et al., 2016; Choi et al., 2017). Team composition, “the nature and attributes of team members” (Guzzo and Dickson, 1996; Jin et al., 2017), is a salient feature in most models. Campion et al. (1996) examined a broad range of theoretical data from past research. They could extract five common themes of team characteristics from past literature. They concluded that “team composition” was included in almost every model of team effectiveness. In the composition of those teams, the articulation of diversity as a feature has attained considerable attention from researchers who consistently report its sterling impact on the effectiveness of team outcomes. Diversity has also been identified as one of the five distinguishing features of the top 100 businesses in the world, according to Fortune magazine’s ranking (Johnstal, 2013; Liang et al., 2015). Since workplaces have become increasingly diverse, it is now imperative to understand how differing viewpoints affect team processes.

### Diversity

Previous literature is observed to have two major theoretical bases for team or group composition. The initial theoretical base stems from “inter-team relations theory” (Watkins and Smith, 2014; Cha et al., 2015; Tao et al., 2016). According to this theory, teams are embedded in a larger social structure. Members of those teams belong to other broader social categories too. They also represent those more significant social categories within their team. Therefore, intra-team interactions can be re-conceptualized as inter-group transactions. Where team refers to organizational team and group is the social category to which people belong. As it was denoted, “interactions between individuals, viewed from an inter-group perspective, reflect the condition of each participant’s group, the relationship of participants to their groups, and the relationship between groups represented by participants as well as their personalities in each ‘interpersonal’ relationship.”

The other theoretical base is rooted in the “social identity perspective” (Cooper et al., 2013; Mols et al., 2015; Hogg, 2016). According to these two theoretical bases, it was argued that people identify and discriminate themselves based on social categories such as gender, age, marital status, etc. In their attempts to achieve a superior social identity, people tend to enhance their self-esteem while challenging the self-esteem of others. As a result, a positive or negative valence is formed between the members of one social group and the members of a different group. Jules (2007) argued that such dual identifications of individuals (one with an organizational team and the other with a social group in society) are represented by two types of components. One is the cognitive component, “categorization of the self into a particular team membership,” and the other is an affective component, “the positive or negative valence attached to that team member.”

Describing the impact of different social and psychological attributes on people’s perceptions about each other and the way they relate to each other matters more than it did in the past. According to early social psychology and organizational behavior researchers, individuals consider only visible physical features when developing perceptions about one another. Iindividuals use these features to categorize and differentiate individuals from others. This form of diversity includes gender, age, marital status, etc. Most of the past researchers labeled this kind of diversity as “demographic diversity” (Richard et al., 2013; Eagly, 2016; Dayan et al., 2017), “social category diversity” (Loyd et al., 2013; Zhou et al., 2015), “visible diversity” (Richard and Miller, 2013), “ascribed diversity” (Blau et al., 1991), “readily detectable diversity” (Lee and Farh, 2004), or “identity groups” (Brickson, 2000).

Another dominant theme in the existing literature is to classify diversity and differentiates it into three distinctive types (Jehn, 1995). The first one is social category diversity, which refers to visible and observable characteristics of an individual, such as their gender and age. The second is named “informational diversity” in literature and refers to the extent to which different individuals possess different knowledge and skills. Both forms are termed surface-level diversity in recent studies. The third type is “value diversity” and refers to differences in individuals’ less visible, concealed attributes. It includes their beliefs, values, personalities, and attitudes that are not easily detectable. This type of diversity is termed deep-level diversity in the current study (Shemla et al., 2016; Lee et al., 2018).

### Conflict

The conflict within teams occurs when an employee observes incompatibilities or discrepancies in others’ personalities, interpersonal styles, perspectives, and ideas (Weingart and Jehn, 2000; Cha et al., 2015; Jiang et al., 2016). Traditionally, conflict is a negative aspect that harms the organization (De Dreu and Weingart, 2003; Dau, 2016). The researchers divide the conflict into two sub-categories; task conflict and relationship conflict. Task conflict is associated with work that permits team members to see the tasks from different viewpoints to improve the team’s outcome (Pelled, 1996; Chen et al., 2019). Hill et al. (2015) refer to relationship conflict as dysfunctional, increasing team members’ anger and frustration. As mentioned in previous studies, this proposition has been empirically tested in previous literature. Still, a meta-analysis by De Dreu and Weingart (2003) and Dinesen et al. (2020) revealed that both kinds of conflict are inversely related to team effectiveness. They are described in a way that the negative influence of relationship conflict is more significant than task conflict. These results can be explained by the notion that task conflict can lead to the instigation of relationship conflict. If it happens, the task-related conflict will also adversely affect teams. In other studies, task and relationship conflict were found to be related in such a way that it was declared that over time, task conflict leads to relationship conflict among members of a team (Simons and Peterson, 2000; Jehn et al., 2015). It sounds reasonable to propose that the evolution of task conflict into relationship conflict makes it detrimental in an indirect way to the performance of a team.

The occurrence of resentment, annoyance, animosity, friction, and interpersonal tensions is due to relationship conflict. The academic literature clearly states that relationship conflict is always negatively linked with individual or group outcomes (Sinha et al., 2016). Consequently, team members may face different challenges such as being given less cognitive resources or time to complete tasks, teammates withholding efforts, distancing themselves from the team, and generally arousing dissatisfaction (Liu et al., 2017; Qu et al., 2017). Task conflict is significantly different from relationship conflict as, in some conditions, it has a positive role in developing team performance and effectiveness. Task conflict is the team’s disagreement of viewpoints and opposing opinions on how to perform a task (Zhang et al., 2015).

### Trust

Trust is a “belief about others’ benevolent motives during a social interaction” (Yamagishi, 2011). The emergence of trust among individuals has been associated with many advantages for the functioning of a team. It is conducive to organizational performance, competitive advantage, better sales, and less employee turnover (Pucetaite et al., 2010; Ford et al., 2017; Wu et al., 2017), etc. More recent literature has focused thoroughly on the role of trust in social interactions at multiple levels (Lount et al., 2012; Ehrke et al., 2020). The concept of interpersonal and inter-group trust is complex. It has been studied from the perspective of different disciplines, i.e., sociology, psychology, management, political, and economic sciences. Experts in these fields agree that trust is a hallmark of an effective relationship.

In an organizational context, researchers also agree that trust is a fundamental ingredient for competitive business advantage and productive working relationships. Based on the work of Lewis and Weigert (2012), trust is divided into two categories: cognitive-based trust and affective-based trust. One is rooted in the cognitive judgments about competence and reliability of other persons (Cognitive Trust), and the other is grounded in effective bonds among people, referred to as “Affective Trust.” In the case of cognitive-based trust between two individuals, the individual chooses whom to trust, and in which respect and under what circumstances they can be trusted. These choices are based on what they perceive as logical, reasonable, and trustworthy (Lewis and Weigert, 2012). On the other hand, affective trust exists in those circumstances where there are emotional ties among people; they invest emotionally in relationships based on trust and convey thoughtfulness and interest in the well-being of the objects of their trust. They have faith in these relationships’ innate goodness and believe such feelings are mutual (Colquitt et al., 2012; De Jong et al., 2021).

Teams are the context within trust emerges, improves, and deteriorates. In team structures, individuals relating to one team mostly share common tasks or goals. Their work is interdependent, and they must accomplish their goals together. Trust among team members can be defined as “employees’ expectations regarding the behavior of their peers,” and the same has been studied in the literature (George et al., 2012; Zheng and Wang, 2021). Trust can be developed at two levels within the same team. Employees can build trust separately with each in a dyadic relationship, or they can cultivate a generalized trust in all team members. The former depends on the other individual’s skills, knowledge, abilities, and personality. The latter kind of generalized trust is developed towards the whole team based on the norms and values of the team. Thus, argued that trust at the team level is not just a mere accumulation or average of all dyadic relationships; instead, it mirrors a member’s beliefs and expectations about other team members.

## Theoretical framework and hypotheses development

In general, understanding the term “diversity” may arouse a negative emotional response from some individuals. The reason is that the term diversity often stirs emotional reactions from people who have associated the word with some negative outcome for them based on any previous incident. As mentioned in the social categorization perspective, people create in-groups and out-groups based on surface-level diversity. Based on those groups, stereotyping and prejudices are developed in a group against other groups (Klein et al., 2011; Shin et al., 2012; Swider et al., 2015). In turn, these prejudices and the resulting anxiety within a team tend to trigger conflict (Roberge and van Dick, 2010; Hollenbeck et al., 2012; Li and Zhu, 2015). It means that a higher level of diversity predicts a higher level of intra-team conflict (Chen et al., 2017; Meng et al., 2018). Based on this, the first hypothesis of the study goes as follows.

**Hypothesis 1:** Diversity (Deep-level and surface-level diversity) will be positively associated with team members’ conflict (task and relationship conflict).

Trust is another emergent state opposite to conflict in its effect and has received considerable attention in the relevant literature (Curşeu and Schruijer, 2010). The emergence of trust among team members has been associated with many advantages for the team’s functioning. In the health sector, where there is high interdependence of work among team members, trusting the judgments of fellow workers or distrusting them can create significant differences in health-related issues or emergencies (Shazi et al., 2015; Cheng et al., 2016). Therefore, it is important to closely look at intra-team trust in the health sector when considering conflict and its causes in the same teams. The two major arguments about the interplay between trust and conflict are that trust among team members reduces conflict and results in beneficial outcomes (Peterson and Behfar, 2003). Second, conflict in a team has a deteriorating effect on the team’s outcome because it erodes trust (Rico et al., 2007; Hsu, 2017). Overall, high levels of intra-team trust are associated with lower levels of conflict among team members (Stahl et al., 2010; Lee and Wong, 2017). Therefore, it is hypothesized that:

**Hypothesis 2:** Intra-Team trust (cognitive trust, affective trust) is negatively associated with intra-team conflict (task conflict and relationship conflict).

Moreover, diversity in this interaction increases the likelihood of the emergence of conflict among team members (Jehn, 1995; Mayo et al., 2017) as opposed to trust, which is more likely to emerge in homogenous (as opposed to diverse) teams (for reference, Curşeu et al., 2008; Nasta et al., 2016). Therefore, while studying diverse teams, the goal should be to minimize the expected conflict by moderating the effect of intra-team trust. Since the current study is to be conducted on diverse teams, conflict is the next variable to relate to diversity. Therefore, the theorized framework considers diversity as an independent variable and conflict as the dependent variable with moderating effect of Trust on the relationship between diversity and conflict.

**Hypothesis 3:** Intra-team trust will moderate the relationship between diversity and conflict such that this relationship will be weaker under circumstances of higher levels of trust among team members.

## Research methodology

### Population

This study’s population was doctors working in public sector hospitals. Public sector hospitals were selected for data collection for several reasons; First, most public hospitals are large hospitals with a complete set of departments and teams working in them, a feature lacking in private hospitals. Second, some private hospitals are too small to have a team-based structure and too specialized to contain any diversity, so private hospitals were not included in the scope of this study. Third, private hospitals did not promise to allow data collection upon request. Finally, there is no reliable source of how many private hospitals are working in Pakistan. There is no reliable base for the classification of those hospitals. As a preliminary investigation, a list of 22 public sector hospitals was extracted from the city government offices. Among those hospitals, three are specialized and are not considered purely public hospitals according to the city district government. Among the remaining 19, 2 did not fulfill the criteria for work settings required by the current study because of their small size; therefore, they were excluded from the sampling frame. Since the unit of analysis was a team, the data was collected from teams of interdependent individuals working together.

### Sampling

Among the 22 identified hospitals, 3 were declared specific to a certain department and not public and eliminated from the sampling frame. Among the remaining 19, two hospitals were too small to be considered. Hospitals with 100 beds or more were selected for this study. Before collecting the data for the main study, a pilot study was conducted by collecting the data from 40 paramedical staff working in different hospitals. The results provide strong support for reliability, discriminant, and convergent validities. For the main study, a simple random sample was drawn, using Microsoft Excel, from the list of 17 hospitals to select 4. Before collecting the data, informed consent was taken from the hospital administration. Hospital management could not provide data on the number of teams working in each hospital. They could provide the data only on the number of departments in each hospital. Therefore, the researcher approached each department to inquire about the number of teams working in each hospital. Approximately, as per our researchers’ best knowledge, 600 teams are working in the selected hospitals. Those teams were also subjected to simple random sampling using MS Excel to draw a sample of 120 teams. The structure of teams was obtained from duty boards displayed in each department or from duty sheets kept by the administrative staff, and questionnaires were distributed among selected team members for data collection.

### Data collection

The unit of analysis for this study is a team. All the variables included in this study were measured at the group level by aggregating individual responses. All the variables—namely diversity, intra-group conflict, and intra-team trust were measured by aggregating the individuals’ responses about their perceptions of the level of each variable in their team. We could aggregate individual responses to the team level because all the variables (diversity, intra-group conflict, and intra-team trust) are only meaningful in a group context. Questions contained phrases relating to “our team” to ask about the shared views of the team members about each variable, keeping the minimum team size to at least three individuals as desired. Teams consisting of two members were dropped.

Self-administered questionnaires were used as a tool for data collection. The administrators in each hospital were approached with an official letter for data collection permission. In some cases, the consent of the head of the department was also required before distributing questionnaires among the team members. The questionnaires were distributed with a request to fill them at the same time in front of the fieldworker, who was supposed to assist in case the respondent found it difficult to understand any part of the questionnaire. Since the data was to be analyzed on the team level, the distribution of questionnaires and collecting them back had to be done carefully so that the questionnaires of one team did not mix with the questionnaires of another team. To keep them separate, unique codes were assigned to each team on temporary bases.

Once the questionnaires were distributed, filled questionnaires were handed back on the same date in some cases. For the remaining questionnaires, there was a follow-up every two days for two weeks. The fieldworker himself visited each team member to personally collect the questionnaire to ensure that data collection occurred distinctly for every team. In total, 700 questionnaires were distributed to individuals across 120 teams. In many follow-ups, teams consistently reported being too busy to respond or having other priorities emerge. Consistent efforts brought 412 filled questionnaires back from 65 teams in total. Four teams were discarded because of an incomplete or inadequate number of questionnaires. As a result, data from 61 teams were included in the final analysis. The response rate at an individual level was 54%, at the team level it was 51%.

### Measures

Self-rated measures were employed using personally administered questionnaire items to measure each variable. The measures for all three variables were adopted from different authors. Special emails were sent to the authors for permission to use the scales. All the measures are group-level measures, but data on those measures were collected from individuals and was aggregated to make it a group-level construct based on the argument put forward by Bresnahan (2008).

A scale developed by Jehn et al. (1999) was used with appropriate modifications to measure deep-level diversity. Jehn (1995) divided diversity into social category, informational, and value diversity. Social categories and informational diversity collectively are termed surface-level diversity based on the work of Harrison et al. (1998) and Jules (2007), and value diversity is deep-level diversity. Deep level diversity was measured on a 5-point Likert scale in which team members were asked to rate how diverse are the individuals in their respective teams based on certain attributes. Surface level diversity includes demographic characteristics such as age, gender, marital status, work experience, and educational qualification. Since the analysis was to take place on a team level, the need was to measure diversity within each team and not across teams. For this purpose, the widely accepted Entropy Based Index (Teachman, 1980) was used. Following is the formula to measure diversity in a team.


Diversity=-Σ⁢Pi⁢log2⁢(Pi)

Where Pi refers to the proportion of the team with each surface-level attribute, four surface-level attributes were considered in this study: age, gender, work experience, and marital status. In the first step, intra-team diversity for each team on each demographic attribute (for example, age) was measured using the abovementioned formula. The result varied on a scale from “0” to “2,” where “0” meant a completely homogenous team and “2” referred to complete heterogeneity. Therefore, if the value is closer to 2, it’s a more heterogeneous team. If the value is closer to 0, it was a more homogenous team concerning that particular attribute (for example, age). Once the diversity of each attribute was measured in a team, an average of all four values was taken to measure total surface-level diversity in one team. That value was used in statistical tests. Two types of conflict (task conflict and relationship conflict) were to be studied. A 4-item, 5-point Likert scale measure originally developed by Jehn (1994) was used to measure each type of conflict. Trust was measured using an instrument developed by Yang (2005), which consisted of 11 items.

## Results

Usable data consisted of 377 questionnaires representing 61 teams in 4 randomly Selected public sector hospitals. Of those 377 individuals, 254 (67%) were men, and the remaining 123 (33%) were women. Respondents belonged to different age groups; 26% were below the age of 25, 34% were between ages 26 and 30, and there were 26% of members between the age of 31 and 35. At the upper end, 9% of respondents were aged between 36 and 40 and only 21 individuals (6%) were above the age of 40. We found 56% of respondents were married, while the remaining 44% were single. Moreover, 74% had work experience ranging from 1 to 5 years, 18% had work experience between 6 and 10 years, and 7% had work experience from 11 to 15 years. These stats also demonstrate that senior members of a team do not possess as much willingness to participate in research projects as the younger members. Descriptive statistics of the study variables are listed in Table 1. As can be seen, all the values for all variables are above 0.70. Therefore, that was no need to eliminate any items from the adopted instrument. The overall trust had the highest value (0.913), and deep-level diversity had the lowest (0.715). The Table 2 provide the details of correlation results.

**TABLE 1:** Descriptive statistics.

Instruments	No. of items	Cronbach’s alpha	Mean
Deep-Level Diversity	4	0.715	−
Task Conflict	4	0.838	2.94
Relationship Conflict	4	0.808	2.63
Cognitive Trust	6	0.885	3.73
Affective Trust	6	0.896	3.29

**TABLE 2:** Correlations.

	RC	TC	CT	AT	Div	Div_Deep	Div_Surface	Trust	Conflict
RC	1.00								
TC	0.55**	1.00							
CT	−0.25*	–0.08	1.00						
AT	−0.34**	−0.39**	0.67**	1.00					
Div	0.15	0.43**	−0.35**	−0.63**	1.00				
Div_Deep	0.11	0.41**	−0.30*	−0.58**	0.82**	1.00			
Div_Surface	0.07	0.38**	–0.06	−0.35**	0.30*	0.42**	1.00		
Trust	−0.33**	−0.26*	0.89**	0.91**	−0.54**	−0.49**	–0.23	1.00	
Conflict	0.83**	0.91**	–0.15	−0.39**	0.35**	0.31*	0.29*	−0.31*	1.00

**Correlation is significant at the 0.01 level (2-tailed).

*Correlation is significant at the 0.05 level (2-tailed).

RC, relationship conflict; TC, task conflict; CT, cognitive trust; AT, affective trust; Div, diversity; Div_Deep, deep l evel diversity; Div_Surface, surface level diversity.

Table 3 summarizes regression analyses for the impact of diversity (and its sub-dimensions) on conflict (and its sub-dimensions). First linear regression analysis was employed to examine the relationship between intra-team diversity and intra-team conflict. The results show that the diversity explained a 12% variance in the dependent variable with significant model statistics. The results also indicated that diversity is significantly and positively associated with task conflict (B = 0.338, *t* = 2.76, *p* = 0.008). For the second and third regression analyses, the results indicated that surface-level diversity explained an 18% variance in task conflict among team members with a positive and significant impact on task conflict (B = 0.442, *t* = 3.786, *p* = 0.000). For deep level diversity, the results showed 18% variance with positive and significant impact on task conflict (B = 0.424, *t* = 3.596, *p* = 0.000). On the other hand, neither surface-level nor deep-level diversity is associated with relationship conflict.

**TABLE 3:** Summaries of regression analyses (diversity and conflict).

	Dependent variable: conflict (composite)			
	
	*R* ^2^	*F*-Value	*B*	*t*-Value
Diversity (composite)	0.125*	8.394*	0.338	2.759*
	
	**Dependent variable: task conflict**			
	
Surface level diversity	0.195*	14.337*	0.442	3.786*
Deep level diversity	0.180*	12.934*	0.424	3.596*
	
	**Dependent variable: relational conflict**			
	
Surface level diversity	0.006	0.327	0.074	0.543
Deep level diversity	0.011	0.656	0.105	0.810

*Significant at 0.01 level.

Table 4 summarizes regression analyses for the impact of intra-team trust (and its sub-dimensions) on intra-team conflict (and its sub-dimensions). The regression analysis results between intra-team trust and intra-team conflict among group members indicated that diversity explained 16% variance with significant model statistics. The statistics also indicated that intra-team trust is significantly and negatively associated with intra-team conflict (B = −0.378, *p* = 0.002). The statistics showed no influence of cognitive trust on task conflict for cognitive trust’s impact on task conflict. On the other hand side, affective-trust explained 18% variance with negative influence (B = −0.428, *p* = 0.001) on task conflict. The regression analysis results of cognitive trust and relationship conflict among team members explained an 8% variance in relationship conflict with a significant but negative impact on relationship conflict (B = −0.294, *p* = 0.05). The effective trust and relationship conflict association result showed that affective trust explained a 21% variance in the dependent variable with a negative impact (B = −0.416, *p* = 0.000) on relationship conflict.

**TABLE 4:** Summaries of regression analyses (trust and conflict).

	Dependent variable: conflict (composite)			
	*R* ^2^	*F*-value	*B*	*t*-Value
Trust (composite)	0.156*	10.917*	−0.378	2.701*
	
	**Dependent variable: task conflict**			
	
Cognitive trust	0.009	0.460	−0.096	−0.743
Affective trust	0.183*	13.259*	−0.428	−3.641*
	
	**Dependent variable: relational conflict**			
	
Cognitive trust	0.086	5.573**	−0.294	−2.361**
Affective trust	0.212	15.901*	−0.416	−3.988*

*Significant at 0.01 level.

**Significant at 0.05 level.

Table 5 reflects the result of moderated regression analysis employed to examine the moderating role of trust on the relationship between diversity and conflict among team members. In the first step, the independent variable (diversity) entered the model with the dependent variable (conflict). The results indicated that diversity explained 12% of the observed variability. In the second step, a moderating variable (trust) was entered into the model with the dependent variable (conflict). The results indicated that diversity explained 8% of the variability. For determining the moderating role of trust, the product of moderator (trust) and independent variable (diversity) was entered in the third step of regression analysis. The moderator explained 8.4% of the variance, whereas the whole model includes independent, and the interaction term (moderator × value diversity) explained 25% of the variability (*R*^2^ = 0.258) in the dependent variable. The results are shown that diversity is directly and positively (*b* = 0.306) associated with conflict.

**TABLE 5:** Moderated regression analysis.

	Dependent variable: conflict (composite)			
	*R* ^2^	*F*-value	*B*	*t*-Value
Diversity	0.125*	7.609*	0.306	2.366**
Trust	0.078*		−0.292	−2.515*
Diversity × trust	0.084*		−0.318	−2.584*

*Significant at 0.01 level.

**Significant at 0.05 level.

Additionally, the moderator and independent variable product term showed a significant but negative relationship (*b* = −0.318) with conflict. The moderation rules suggested that the impact of the product term consisted of the moderator (trust) and independent variable (value diversity) having a significant impact on the dependent variables when moderation existed. The significance of interaction term is higher as compared to the moderator itself. Therefore the statistics suggest that increased diversity among team members leads to increased conflict among them. Still, in cases where individuals with diverse skills and attitudes also trust the competencies of fellow members, it eliminates the chances for the emergence of conflict in those teams.

## Discussion and conclusion

The results of this study reflected that with the increase in diversity, conflict among team members would increase too. These results are consistent with the findings of Agar (2002), who studied the relationship between different types of diversity with different types of conflict. The results also affirmed an association between the dimensions of diversity (surface level and deep level diversity) and the dimensions of conflict (task conflict and relationship conflict). Due to surface-level diversity, the change in relationship conflict is lesser than the change in task conflict. On the other hand, it had been hypothesized in the study that deep level diversity is responsible for a more significant change in relationship conflict and comparatively a smaller change in task conflict. Results, however, disclosed that the change in task conflict due to deep level diversity is more significant than the change in relationship conflict. Moreover, like the mechanisms of the diversity-conflict relationship, the dimensions of team trust and conflict intermingle similarly. It was hypothesized that the cognitive component of trust has a more substantial impact on task-related conflict and a lesser impact on relationship-related conflict.

First, it is worth mentioning that different forms of diversity and trust collectively are responsible for significant variation in conflict among team members. Overall diversity was also found to be impacting the level of conflict among team members. These findings are consistent with those of de Wit et al. (2012) Findings of this study go in line with the proponents of deep-level diversity (sometimes referred to as “Cognitive Diversity”) in suggesting that the two surface and deep levels of diversity have different impacts on the functioning of teams. When studied differently, the results of each type of diversity showed that different forms of diversity impact conflict differently. It is possible that a group that seems homogeneous on surface attributes may yet possess significant diversity at a deeper level.

Similarly, the results reaffirm the already established theory of bi-dimensionality of conflict that has already been proved by different researchers (Jehn et al., 1999; Agar, 2002; Choi and Sy, 2010) who differentiate conflict as task and relationship related. The hypotheses relevant to the positive association between diversity and conflict were significantly supported and the results, were consistent with existing literature. Results are consistent with the findings of previous studies in supporting that people differentiate and stereotype each other based on surface-level attributes, and these biased feelings about each other harm their progress on the task. However, the proposition that deep-level diversity causes more relationship conflict and less task conflict among people was not supported after analyzing the data. The findings were exactly the opposite of what was proposed. Although this finding is consistent with some findings in the literature (Jehn et al., 1997, 1999) and is against the findings of some other studies, such as Parayitam et al. (2012), a more rigorous study of more recent literature provides a better insight into the reason for this finding.

Advocates of deep-level diversity claim that since this diversity exists at the cognition level, more deep-level diversity creates better awareness about alternative courses of action while working on a task (Kilduff et al., 2000). More knowledge among diverse individuals attracts in-depth discussions related to the task’s content, which leads to more task conflict. Therefore, deep-level diversity, like surface-level diversity, is associated more with task conflict than relationship conflict. These findings can be attributed to the presence of trust among team members, which is discussed later. While examining the interrelatedness between the dimension of trust and dimensions of conflict, it was proposed that cognitive trust hurts conflict. The dimensions of conflict intermingle with the dimensions of trust such that its impact on task conflict is more than its impact on relationship conflict. This proposition was also rejected after the analysis of data. These results are against those of Yang (2005).

This puzzling finding embraces a compelling justification when we consider the study’s context. The study was conducted on teams working in public hospitals consisting primarily of doctors, which is a highly suitable population for studying dynamics and interplay of diversity, conflict, and trust. Doctors possess highly specified knowledge and precise expertise in their specific fields. Moreover, since they deal with life and death situations, they realize the high stakes of their decision and act accordingly. It has been verified that diversity is more effective when team members possess more task-relevant information (Van Knippenberg et al., 2004). Doctors also possess high task-relevant knowledge; hence there is more chance of exchange of information which includes discussing and integrating information relevant to a task at hand. This shared confidence will encourage them to share their point of view more openly without the fear that the other person may feel attacked. Therefore, when there are more discussions on issues in the presence of higher trust, there will be more task conflict, but that task conflict will always be functional unless the trust deteriorates (Olson et al., 2007). Doctors realize the high stakes of their decisions and do not let the task conflict derail them from making the best possible decision.

A diverse team in a hospital contains members who affirm their points of view and challenge the opinions of others. In the presence of trust, members push each other to greater heights knowing that increased task conflict is beneficial for quality solutions. On the other hand, members of the teams with lower levels of trust respond with more “professional courtesy” rather than challenging others. This “nicety” discourages them from sharing their opinions openly hence reducing the chances of a conflict. Moreover, the inclusion of trust among those team members does help resolve such matters to an operational cause. Finally, the proposed moderating role of trust in the relationship was also supported with the help of received data. It was proved that Intra-team trust moderates the relationship between diversity and conflict such that this relationship will be weaker under higher levels of trust among team members.

### Practical implications

This study will benefit organizational behavior theory and practice in four ways. First of all, the findings of the current study refine the existing knowledge about the moderating effect of trust on the relationship between diversity and conflict at the group level, as previously there was little interest in existing literature when considering emergent states of the same moderating relationship. The extant literature also possesses a gap regarding the interplay among the dimensions of trust, conflict, and diversity in team members. This model goes beyond the team process and considers emergent states in team dynamics. Secondly, this study focuses on an individual level, and their responses will be aggregated to make it onto the group level for two reasons. *One*, this study considers diversity in the perceptions of individuals. The notion of perceptual diversity calls for considering group members’ responses individually because homogenous individuals have heterogeneous perceptions. *Two*, the experience of conflict also lies in person-to-person relationships, so it is better to focus on an individual level and aggregate their responses to the group level. Because “teams do not think and feel, individuals do” (Korsgaard et al., 2008).

In the work settings of Pakistan, this study’s findings can change how managers view the management of conflict among work teams. This study contributes to the literature by highlighting the role of intra-team trust in managing conflict among team members. At first, this study differentiates between the two types of conflict and assures their distinctiveness. This attempt also clarifies the claim of the dysfunctionality of both types of conflict (De Dreu and Weingart, 2003) by clearly highlighting the functionality of task conflict in the presence of trust among team members. Therefore, managers must realize that promoting intra-team trust is more important than avoiding intra-team conflict. Secondly, the findings of this study present managers with an awareness and understanding of deep-level diversity. It is already established theory that diversity is coupled with several worthy outcomes, but can cause conflict as one of its destructive consequences. Mostly, the managers take into account surface-level attributes and count on demographic features when managing team diversity (Agar, 2002). This study offers a more profound insight by stressing the concealed deep-level diversity that can solely be responsible for heterogeneity among homogeneous team members. According to the findings of this study, no category of diversity should be ignored since each type impacts conflict separately and together. It might surprise managers that cognitive trust alone does not guarantee mitigating task-related conflict. Instead, it enhances task conflict. This is not a discouraging or confusing finding but a reminder of the valuable role of trust, which transforms the task conflict into a worthy consequence, as explained in detail.

### Strengths and limitations

We want to mention the specific methodological strengths of this study before discussing its limitations. First, this study was conducted in a hospital setting, and most of the data were collected from highly educated and extremely trained doctors. They have a specialized but different set of knowledge and skills in the same team, making it diverse. Their work and tasks are highly interdependent, they have to face crucial challenges in emergencies, which may cause a stressful working climate, and they have to deal with other human beings in their work. Therefore, it is highly likely that conflict may arise among such teams (Studdert et al., 2003). On the other hand, because of the high stakes of their decisions and their specialized skills, they realize that they cannot let the conflict deteriorate the quality of their decision. Therefore, they must make conflict a valuable feature of their teams (Olson et al., 2007).

Secondly, the study is conducted on a team level, which is very rare in studies related to trust-conflict relationships. Conflict and trust are group-related phenomena, meaning they cannot occur in a single individual; it takes more than one individual to study conflict, trust, and diversity. Therefore, team level is another strength of the current study. Moreover, according to the researcher’s best knowledge, no study in Pakistan has measured diversity within a team and not among all individuals in a sample. An Entropy-Based Index was utilized to accomplish this task, contributing to encouraging efforts to promote quality and reliable research in Pakistan. Despite these strengths, this study is not perfect in every aspect and should be appreciated only within its limitations. We shall mention the particular methodological limitations of this study. First, studying the two types of conflict required a very sophisticated longitudinal design because it was established by Peterson and Behfar (2003) that one type of conflict leads to the other type (over time, task conflict leads to relationship conflict), whereas the second is cross-sectional research design, due to which the results should be carefully interpreted.

## Data availability statement

The raw data supporting the conclusions of this article will be made available by the authors, without undue reservation.

## Ethics statement

Ethical review and approval was not required for the study on human participants in accordance with the local legislation and institutional requirements. The patients/participants provided their written informed consent to participate in this study.

## Author contributions

All authors listed have made a substantial, direct, and intellectual contribution to the work, and approved it for publication.

## References

[B1] Abott M. (2010). *The Effect of Experiential Diversity on Conflict in Top Management Teams of Venture Firms.* Cleveland, OH: Case Western Reserve University.

[B2] Agar F. P. (2002). *Diversity, Conflict, and Systems Leadership in Project Groups: a Longitudinal Study.* Lubbock, TX: Texas Tech University.

[B3] Baker M. J. Détienne F. Barcellini F. (2017). “Argumentation and conflict management in online epistemic communities: a narrative approach to wikipedia debates,” in *Interpersonal Argumentation in Educational and Professional*, eds Contexts F. Bova A. (Cham: Springer). 10.1007/978-3-319-59084-4_7

[B4] Bamel U. K. Paul H. Bamel N. (2018). *Managing Workplace Diversity Through Organizational Climate in Flexibility in Resource Management.* Singapore: Springer. 10.1007/978-981-10-4888-3_6

[B5] Behfar K. Friedman R. Brett J. (2016). Managing co-occurring conflicts in teams. *Group Decision Negotiation* 25 501–536. 10.1007/s10726-015-9450-x

[B6] Bell S. T. (2007). Deep-level composition variables as predictors of team performance: a meta- analysis. *J. Appl. Psychol.* 92:595. 10.1037/0021-9010.92.3.595 17484544

[B7] Blau P. M. Ruan D. Ardelt M. (1991). Interpersonal choice and networks in China. *Soc. Forces* 69 1037–1062. 10.2307/2579301

[B8] Bresnahan C. G. (2008). *Attachment style as a predictor of group conflict, post-conflict relationship repair, trust and leadership style.* Los Angeles, CA: University of Southern California.

[B9] Breuer C. Hüffmeier J. Hertel G. (2016). Does trust matter more in virtual teams? A meta-analysis of trust and team effectiveness considering virtuality and documentation as moderators. *J. Appl. Psychol.* 101:1151. 10.1037/apl0000113 27228105

[B10] Brickson S. (2000). The impact of identity orientation on individual and organizational outcomes in demographically diverse settings. *Acad. Manag. Rev.* 25 82–101. 10.2307/259264

[B11] Campion M. A. Papper E. M. Medsker G. J. (1996). Relations between work team characteristics and effectiveness: a replication and extension. *Personnel Psychol.* 49 429–452. 10.1111/j.1744-6570.1996.tb01806.x

[B12] Cha J. Kim Y. Lee J.-Y. Bachrach D. G. (2015). Transformational leadership and inter-team collaboration: exploring the mediating role of teamwork quality and moderating role of team size. *Group Organ. Manag.* 40 715–743. 10.1177/1059601114568244

[B13] Chen X. Liu J. Zhang H. Kwan H. K. (2019). Cognitive diversity and innovative work behaviour: the mediating roles of task reflexivity and relationship conflict and the moderating role of perceived support. *J. Occup. Organ. Psychol.* 92 671–694. 10.1111/joop.12259

[B14] Chen Z. Zhu J. Zhou M. (2017). “Disagree in disagreement: How does conflict asymmetry affect team outcomes,” in *Proceedings of the Academy of Management* (Briarcliff Manor, NY: Academy of Management). 10.5465/AMBPP.2017.13142abstract

[B15] Cheng X. Fu S. Druckenmiller D. (2016). Trust development in globally distributed collaboration: a case of US and Chinese mixed teams. *J. Manag. Inform. Systems* 33 978–1007. 10.1080/07421222.2016.1267521

[B16] Choi J. N. Sy T. (2010). Group-level organizational citizenship behavior: Effects of demographic faultlines and conflict in small work groups. *J. Org. Behav.* 31 1032–1054.

[B17] Choi S. B. Kim K. Kang S. W. (2017). Effects of transformational and shared leadership styles on employees’ perception of team effectiveness. *Soc. Behav. Pers.* 45 377–386. 10.1111/jmwh.12707 29377534

[B18] Colquitt J. A. LePine J. A. Piccolo R. F. Zapata C. P. Rich B. L. (2012). Explaining the justice–performance relationship: trust as exchange deepener or trust as uncertainty reducer? *J. Appl. Psychol.* 97:1. 10.1037/a0025208 21910516

[B19] Cooper D. Patel P. C. Thatcher S. M. (2013). It depends: environmental context and the effects of faultlines on top management Team performance. *Organ. Sci.* 25 633–652. 10.1287/orsc.2013.0855 19642375

[B20] Costa P. L. Passos A. M. Bakker A. B. (2015). Direct and contextual influence of team conflict on team resources, team work engagement, and team performance. *Negotiation Conflict Manag. Res.* 8 211–227. 10.1111/ncmr.12061

[B21] Curşeu P. L. Schalk R. Wessel I. (2008). How do virtual teams process information? A literature review and implications for management. *J. Manag. Psychol.* 23, 628–652. 10.1108/02683940810894729

[B22] Curşeu P. L. Schruijer S. G. (2010). Does conflict shatter trust or does trust obliterate conflict? revisiting the relationships between team diversity, conflict, and trust. *Group Dynamics: Theory Res. Practice* 14:66. 10.1037/a0017104

[B23] Dau L. A. (2016). Biculturalism, team performance, and cultural-faultline bridges. *J. Int. Manag.* 22 48–62. 10.1016/j.intman.2015.10.001

[B24] Dayan R. Heisig P. Matos F. (2017). Knowledge management as a factor for the formulation and implementation of organization strategy. *J. Knowl. Manage.* 21 308–329.

[B25] De Dreu C. K. Weingart L. R. (2003). Task versus relationship conflict, team performance, and team member satisfaction: a meta-analysis. *J. Appl. Psychol.* 88:741. 10.1037/0021-9010.88.4.741 12940412

[B26] De Jong B. Gillespie N. Williamson I. Gill C. (2021). Trust consensus within culturally diverse teams: a multistudy investigation. *J. Manag.* 47 2135–2168. 10.1177/0149206320943658

[B27] de Wit F. R. Greer L. L. Jehn K. A. (2012). The paradox of intragroup conflict: a meta- analysis. *J. Appl. Psychol.* 97:360. 10.1037/a0024844 21842974

[B28] Dinesen P. T. Schaeffer M. Sønderskov K. M. (2020). Ethnic diversity and social trust: a narrative and meta-analytical review. *Ann. Rev. Political Sci.* 23 441–465. 10.1146/annurev-polisci-052918-020708

[B29] Dolmans D. Michaelsen L. Van Merrienboer J. van der Vleuten C. (2015). Should we choose between problem-based learning and team-based learning? no, combine the best of both worlds! *Med. Teach.* 37 354–359. 10.3109/0142159X.2014.948828 25154342

[B30] Eagly A. H. (2016). When passionate advocates meet research on diversity, does the honest broker stand a chance? *J. Soc. Issues* 72 199–222.

[B31] Ehrke F. Bruckmüller S. Steffens M. C. (2020). A double-edged sword: how social diversity affects trust in representatives via perceived competence and warmth. *Eur. J. Soc. Psychol.* 50 1540–1554. 10.1002/ejsp.2709

[B32] Ford R. C. Piccolo R. F. Ford L. R. (2017). Strategies for building effective virtual teams: trust is key. *Bus. Horizons* 60 25–34. 10.1016/j.bushor.2016.08.009

[B33] George E. Chattopadhyay P. Zhang L. L. (2012). Helping hand or competition? the moderating influence of perceived upward mobility on the relationship between blended workgroups and employee attitudes and behaviors. *Organ. Sci.* 23 355–372. 10.1287/orsc.1100.0606 19642375

[B34] Guzzo R. A. Dickson M. W. (1996). Teams in organizations: recent research on performance and effectiveness. *Annu. Rev. Psychol.* 47 307–338. 10.1146/annurev.psych.47.1.307 15012484

[B35] Harrison D. A. Price K. H. Bell M. P. (1998). Beyond relational demography: time and the effects of surface-and deep-level diversity on work group cohesion. *Acad. Manag. J.* 41 96–107. 10.5465/256901 256901

[B36] Hawkins S. (2016). *The Long Arc of Diversity Bends Towards Equality: Deconstructing the Progressive Critique of Workplace Diversity Efforts.* Available online at: https://ssrn.com/abstract=2954100 (accessed July 10, 2021).

[B37] Held C. M. (2017). *Conflict Response Teams: a New Method of Mediation.* Muncie, IN: Ball State University.

[B38] Hill K. Chênevert D. Poitras J. (2015). Changes in relationship conflict as a mediator of the longitudinal relationship between changes in role ambiguity and turnover intentions. *Int. J. Conflict Manag.* 26 44–67. 10.1108/IJCMA-11-2013-0091

[B39] Hitt M. A. Biermant L. Shimizu K. Kochhar R. (2001). Direct and moderating effects of human capital on strategy and performance in professional service firms: a resource- based perspective. *Acad. Manag. J.* 44 13–28. 10.2307/3069334

[B40] Hjerto K. B. Kuvaas B. (2017). Burning hearts in conflict: new perspectives on the intragroup conflict and team effectiveness relationship. *Int. J. Conflict Manag.* 28 50–73. 10.1108/IJCMA-02-2016-0009

[B41] Hogg M. A. (2016). “Social identity theory,” in *Understanding peace and conflict through social identity theory*, eds Ferguson N. Haji R. McKeown S. (Cham: Springer), 3–17.

[B42] Hollenbeck J. R. Beersma B. Schouten M. E. (2012). Beyond team types and taxonomies: a dimensional scaling conceptualization for team description. *Acad. Manag. Rev.* 37 82–106. 10.5465/armr.2010.0181

[B43] Hollenbeck J. R. DeRue D. S. Guzzo R. (2004). Bridging the gap between I/O research and HR practice: improving team composition, team training, and team task design. *Hum. Resource Manag.* 43 353–366. 10.1002/hrm.20029

[B44] Hsu Y. (2017). Work values, conflict, and team cooperation among engineering designers. *J. Eng. Design* 28 799–820. 10.1111/hex.13499 35472122 PMC9327859

[B45] Hu N. Wu J. Gu J. (2019). Cultural intelligence and employees’ creative performance: the moderating role of team conflict in interorganizational teams. *J. Manag. Organ.* 25, 96–116. 10.1017/jmo.2016.64

[B46] Jehn K. A. (1994). Enhancing effectiveness: an investigation of advantages and disadvantages of value-based intragroup conflict. *Int. J. Conflict Manag.* 5 223–238. 10.1108/eb022744

[B47] Jehn K. A. (1995). A multimethod examination of the benefits and detriments of intragroup conflict. *Administrative Sci. Quarterly* 40 256–282. 10.2307/2393638

[B48] Jehn K. A. Chadwick C. Thatcher S. M. B. (1997). To agree or not to agree: The effects of value congruence, individual demographic dissimilarity, and conflict on workgroup outcomes. *Int. J. Confl. Manage.* 8 287–305.

[B49] Jehn K. A. De Wit F. R. Barreto M. Rink F. (2015). Task conflict asymmetries: Effects on expectations and performance. *Int. J. Confl. Manage.* 26 172–191.

[B50] Jehn K. A. Northcraft G. B. Neale M. A. (1999). Why differences make a difference: a field study of diversity, conflict and performance in workgroups. *Administrative Sci. Quarterly* 44 741–763. 10.2307/2667054

[B51] Jiang X. Flores H. R. Leelawong R. Manz C. C. (2016). The effect of team empowerment on team performance: a cross-cultural perspective on the mediating roles of knowledge sharing and intra-group conflict. *Int. J. Conflict Manag.* 27 62–87. 10.1108/IJCMA-07-2014-0048

[B52] Jin L. Madison K. Kraiczy N. D. Kellermanns F. W. Crook T. R. Xi J. (2017). Entrepreneurial team composition characteristics and new venture performance: a meta-analysis. *Entrepreneurship Theory Practice* 41 743–771.

[B53] Johnstal S. P. (2013). Successful strategies for transfer of learned leadership. *Perform. Improvement* 52 5–12. 10.1002/pfi.21358

[B54] Jules C. (2007). *Diversity of Member Composition and Team Learning in Organizations.* Cleveland, OH: Case Western Reserve University.

[B55] Kilduff M. Angelmar R. Mehra A. (2000). Top management-team diversity and firm performance: examining the role of cognitions. *Organ. Sci.* 11 21–34. 10.1287/orsc.11.1.21.12569 19642375

[B56] Klein K. J. Knight A. P. Ziegert J. C. Lim B. C. Saltz J. L. (2011). When team members’ values differ: the moderating role of team leadership. *Organ. Behav. Human Decision Proc.* 114 25–36. 10.3310/hta22100 29457585 PMC6485678

[B57] Korsgaard M. A. Jeong S. S. Mahony D. M. Pitariu A. H. (2008). A multilevel view of intragroup conflict. *J. Manag.* 34 1222–1252. 10.1177/0149206308325124

[B58] Lee C. Farh J. L. (2004). Joint effects of group efficacy and gender diversity on group cohesion and performance. *Appl. Psychol.* 53 136–154. 10.1111/j.1464-0597.2004.00164.x

[B59] Lee C. Wong C. S. (2017). The effect of team emotional intelligence on team process and effectiveness. *J. Manag. Organ.* 25 1–16. 10.1017/jmo.2017.43

[B60] Lee H. W. Choi J. N. Kim S. (2018). Does gender diversity help teams constructively manage status conflict? an evolutionary perspective of status conflict, team psychological safety, and team creativity. *Organ. Behav. Hum. Decision Proc.* 144 187–199. 10.1016/j.obhdp.2017.09.005

[B61] Lee S. K. J. Yu K. (2004). Corporate culture and organizational performance. *J. Managerial Psychol.* 19 340–359. 10.1108/02683940410537927

[B62] Lewis J. D. Weigert A. J. (2012). The social dynamics of trust: theoretical and empirical research, 1985-2012. *Soc. Forces* 91 25–31. 10.1093/sf/sos116 32080734

[B63] Li C.-R. Li C.-X. Lin C.-J. Liu J. (2018). The influence of team reflexivity and shared meta-knowledge on the curvilinear relationship between team diversity and team ambidexterity. *Manag. Decision* 56 1033–1050. 10.1108/MD-05-2017-0522

[B64] Li J. Zhu J. (2015). “Does self-verification striving benefit expertise recognition? examing team conflict as a moderator,” in *Proceedings of the Academy of Management* (Briarcliff Manor, NY: Academy of Management). 10.5465/ambpp.2015.15940abstract

[B65] Li J. Jehn K. Roe R. (2016). The predictors and consequences of within-team conflict change: a dynamic mediation model. *Int. J. Psychol.* 51:764.

[B66] Liang H.-Y. Shih H.-A. Chiang Y.-H. (2015). Team diversity and team helping behavior: the mediating roles of team cooperation and team cohesion. *Eur. Manag. J.* 33 48–59. 10.1016/j.emj.2014.07.002

[B67] Liu Z. Deng C.-J. Wu B. Ge L. (2017). “Workplace conflict, status-conferral criteria and job performance: status competition perspective,” in *Paper presented at the Academy of Management Proceedings* (Briarcliff Manor, NY).

[B68] Löhr K. Weinhardt M. Graef F. Sieber S. (2017). Enhancing communication and collaboration in collaborative projects through conflict prevention and management systems. *Organ. Dynamics* 47 259–264. 10.1016/j.orgdyn.2017.10.004

[B69] Lount R. B. Jr. Sheldon O. J. Rink F. Phillips K. W. Lount R. B. Jr. (2012). “How much relationship conflict really exists? biased perceptions of racially diverse teams,” in *Paper presented at the Annual Interdisciplinary Network for Group Research Conference* (Chicago, IL). 10.2139/ssrn.2120006

[B70] Loyd D. L. Wang C. S. Phillips K. W. Lount R. B. Jr. (2013). Social category diversity promotes premeeting elaboration: the role of relationship focus. *Organ. Sci.* 24 757–772. 10.1287/orsc.1120.0761 19642375

[B71] Marks M. A. Mathieu J. E. Zaccaro S. J. (2001). A temporally based framework and taxonomy of team processes. *Acad. Manag. Rev.* 26 356–376. 10.5465/amr.2001.4845785

[B72] Mayo M. Kakarika M. Mainemelis C. Deuschel N. T. (2017). A metatheoretical framework of diversity in teams. *Hum. Relations* 70 911–939. 10.1177/0018726716679246

[B73] McGrath J. E. (1964). Toward a “theory of method” for research on organizations. *New Perspect. Organ. Res.* 533:556.

[B74] Meng A. Clausen T. Borg V. (2018). The association between team-level social capital and individual-level work engagement: differences between subtypes of social capital and the impact of intra-team agreement. *Scand. J. Psychol.* 59 198–205. 10.1111/sjop.12435 29460370

[B75] Mols F. Haslam S. A. Jetten J. Steffens N. K. (2015). Why a nudge is not enough: A social identity critique of governance by stealth. *Eur. J. Polit. Res.* 54 81–98.

[B76] Mor Barak M. E. (2016). *Managing Diversity: Toward a Globally Inclusive Workplace.* Thousand Oaks, CA: Sage Publications.

[B77] Muldoon J. (2017). The Hawthorne studies: an analysis of critical perspectives, 1936-1958. *J. Manag. History* 23 74–94. 10.1108/JMH-09-2016-0052

[B78] Nasta L. Pirolo L. Wikström P. (2016). Diversity in creative teams: a theoretical framework and a research methodology for the analysis of the music industry. *Creat. Industries J.* 9 97–106. 10.1080/17510694.2016.1154653

[B79] Ng J. Leonardi P. Contractor N. (2017). Teaming at the limit: enhancing team effectiveness with enterprise social media affordances. *Acad. Manag. Ann. Meet. Proc.* 2017:13836. 10.5465/AMBPP.2017.13

[B80] Olson B. J. Parayitam S. Bao Y. (2007). Strategic decision making: the effects of cognitive diversity, conflict, and trust on decision outcomes. *J. Manag.* 33 196–222.

[B81] Parayitam S. Olson B. J. Bao Y. (2012). Effects of cognitive diversity on relationship conflict, agreement–seeking behaviour and decision quality: a study of Chinese management teams. *Int. J. Chinese Culture Manag.* 3 174–187. 10.1504/IJCCM.2012.046031 35009967

[B82] Pelled L. H. (1996). Demographic diversity, conflict, and work group outcomes: an intervening process theory. *Organ. Sci.* 7 615–631. 10.1287/orsc.7.6.615 19642375

[B83] Peterson R. S. Behfar K. J. (2003). The dynamic relationship between performance feedback, trust, and conflict in groups: a longitudinal study. *Organ. Behav. Hum. Decision Proc.* 92 102–112. 10.1016/S0749-5978(03)00090-6

[B84] Pretty J. Adams B. Berkes F. de Athayde S. F. Dudley N. Hunn E. (2009). The intersections of biological diversity and cultural diversity: towards integration. *Conservation Soc.* 7 100–112. 10.4103/0972-4923.58642

[B85] Pucetaite R. Lämsä A.-M. Novelskaite A. (2010). Building organizational trust in a low- trust societal context. *Baltic J. Manag.* 5 197–217.

[B86] Qu Y. (2017). *Disentangling the Effects of Abusive Supervision on Employee Creativity: The Role of Affect, Mindfulness, and Team Conflict.* Doctor of Philosophy Ph.D. thesis, Coral Gables, FL: University of Miami.

[B87] Qu Y. Todorova G. Dasborough M. Shi Y. (2017). The effects of abusive supervision on team task conflict and relationship conflict. *Acad. Manag. Ann. Meet. Proc.* 2017:12653.

[B88] Rego A. Owens B. Yam K. C. Bluhm D. Cunha M. P. E. Silard A. (2019). Leader humility and team performance: exploring the mediating mechanisms of team PsyCap and task allocation effectiveness. *J. Manag.* 45, 1009–1033. 10.1177/0149206316688941

[B89] Richard O. C. Miller C. D. (2013). “Considering diversity as a source of competitive advantage in organizations,” in *The Oxford Handbook of Diversity and Work*, ed. Roberson Q. M. (Oxford: Oxford University Press), 239–250. 10.1093/oxfordhb/9780199736355.013.0014

[B90] Richard O. C. Kirby S. L. Chadwick K. (2013). The impact of racial and gender diversity in management on financial performance: how participative strategy making features can unleash a diversity advantage. *Int. J. Hum. Resource Manag.* 24 2571–2582. 10.1080/09585192.2012.744335

[B91] Rico R. Molleman E. Sánchez-Manzanares M. Van der Vegt G. S. (2007). The effects of diversity faultlines and team task autonomy on decision quality and social integration. *J. Manag.* 33 111–132. 10.1177/0149206306295307

[B92] Roberge M. -É van Dick R. (2010). Recognizing the benefits of diversity: when and how does diversity increase group performance? *Hum. Resource Manag. Rev.* 20 295–308.

[B93] Scott M. Jacob A. Hendrie D. Parsons R. Girdler S. Falkmer T. (2017). Employers’ perception of the costs and the benefits of hiring individuals with autism spectrum disorder in open employment in Australia. *PLoS One* 12:e0177607. 10.1371/journal.pone.0177607 28542465 PMC5436808

[B94] Selzer V. L. Schumann J. H. Büttgen M. Ates Z. Komor M. Volz J. (2021). Effective coping strategies for stressed frontline employees in service occupations: outcomes and drivers. *Service Industries J.* 41 382–399. 10.1080/02642069.2018.1548613

[B95] Shazi R. Gillespie N. Steen J. (2015). Trust as a predictor of innovation network ties in project teams. *Int. J. Project Manag.* 33 81–91. 10.1016/j.ijproman.2014.06.001

[B96] Shemla M. Meyer B. Greer L. Jehn K. A. (2016). A review of perceived diversity in teams: Does how members perceive their team’s composition affect team processes and outcomes? *J. Org. Behav.* 37 S89–S106.

[B97] Shin S. J. Kim T.-Y. Lee J.-Y. Bian L. (2012). Cognitive team diversity and individual team member creativity: a cross-level interaction. *Acad. Manag. J.* 55 197–212. 10.5465/amj.2010.0270

[B98] Simons T. L. Peterson R. S. (2000). Task conflict and relationship conflict in top management teams: the pivotal role of intragroup trust. *J. Appl. Psychol.* 85:102. 10.1037/0021-9010.85.1.102 10740960

[B99] Sinha R. Janardhanan N. S. Greer L. L. Conlon D. E. Edwards J. R. (2016). Skewed task conflicts in teams: what happens when a few members see more conflict than the rest? *J. Appl. Psychol.* 101:1045. 10.1037/apl0000059 26949822

[B100] Stahl G. K. Maznevski M. L. Voigt A. Jonsen K. (2010). Unraveling the effects of cultural diversity in teams: a meta-analysis of research on multicultural work groups. *J. Int. Bus. Stud.* 41 690–709. 10.1057/s41267-020-00389-9 33487775 PMC7812115

[B101] Studdert D. M. Mello M. M. Burns J. P. Puopolo A. L. Galper B. Z. Truog R. D. (2003). Conflict in the care of patients with prolonged stay in the ICU: Types, sources, and predictors. *Intensive Care Med.* 29 1489–1497. 12879243 10.1007/s00134-003-1853-5

[B102] Swider B. W. Zimmerman R. D. Charlier S. D. Pierotti A. J. (2015). Deep-level and surface-level individual differences and applicant attraction to organizations: a meta-analysis. *J. Vocational Behav.* 88 73–83.

[B103] Tao Y.-L. Chuang H.-L. Lin E. S. (2016). Compensation and performance in Major League Baseball: evidence from salary dispersion and team performance. *Int. Rev. Econ. Finance* 43 151–159. 10.1016/j.iref.2015.10.037

[B104] Tasheva S. Hillman A. (2018). Integrating diversity at different levels: multi-level human capital, social capital, and demographic diversity and their implications for team effectiveness. *Acad. Manag. Rev. AMR* 2015:0396.

[B105] Teachman J. D. (1980). Analysis of population diversity measures of qualitative variation. *Sociol. Methods Res.* 8 341–362. 10.1177/004912418000800305

[B106] Van Knippenberg D. De Dreu C. K. Homan A. C. (2004). Work group diversity and group performance: an integrative model and research agenda. *J. Appl. Psychol.* 89:1008. 10.1037/0021-9010.89.6.1008 15584838

[B107] Watkins M. B. Smith A. N. (2014). Importance of women’s political skill in male- dominated organizations. *J. Managerial Psychol.* 29:6. 10.1108/JMP-06-2012-0106

[B108] Weingart L. Jehn K. A. (2000). “Manage intra-team conflict through collaboration,” in *Handbook of Principles of Organizational Behavior*, ed. Locke E. A. (Hoboken, NJ: John Wiley & Sons, Ltd.), 226–238.

[B109] Wu G. Zhao X. Zuo J. (2017). Relationship between project’s added value and the trust–conflict interaction among project teams. *J. Manag. Eng.* 33:04017011. 10.1061/(ASCE)ME.1943-5479.0000525 29515898

[B110] Yamagishi S. K. A. (2011). *Microbial Biodiversity and Biogeography on the Deep Seafloor, Changing Diversity in Changing Environment.* PhD. Oscar Grillo thesis, London: InTech.

[B111] Yang Y. (2005). Can the strengths of AIC and BIC be shared? A conflict between model indentification and regression estimation. *Biometrika* 92 937–950.

[B112] Zhang X.-A. Li N. Ullrich J. van Dick R. (2015). Getting everyone on board: the effect of differentiated transformational leadership by CEOs on top management team effectiveness and leader-rated firm performance. *J. Manag.* 41 1898–1933. 10.1177/0149206312471387

[B113] Zheng Q. Wang L. (2021). Teammate conscientiousness diversity depletes team cohesion: the mediating effect of intra-team trust and the moderating effect of team coaching. *Curr. Psychol.* 10.1007/s12144-021-01946-7

[B114] Zhou W. Vredenburgh D. Rogoff E. G. (2015). Informational diversity and entrepreneurial team performance: Moderating effect of shared leadership. *Int. Entrep. Manage. J.* 11 39–55.

